# Low Serum Potassium Levels Increase the Infectious-Caused Mortality in Peritoneal Dialysis Patients: A Propensity-Matched Score Study

**DOI:** 10.1371/journal.pone.0127453

**Published:** 2015-06-19

**Authors:** Silvia Carreira Ribeiro, Ana Elizabeth Figueiredo, Pasqual Barretti, Roberto Pecoits-Filho, Thyago Proenca de Moraes, all centers that contributed to the BRAZPD II study

**Affiliations:** 1 School of Medicine, Pontifícia Universidade Católica do Paraná (PUCPR), Curitiba, Brazil; 2 Graduate Program in Medicine and Health Sciences, Pontifícia Universidade Católica do Rio Grande do Sul (PUCRS), Porto Alegre, Brazil; 3 School of Medicine, UNESP, Botucatu, Brazil; Hospital Universitario de La Princesa, SPAIN

## Abstract

**Background and Objectives:**

Hypokalemia has been consistently associated with high mortality rate in peritoneal dialysis. However, studies investigating if hypokalemia is acting as a surrogate marker of comorbidities or has a direct effect in the risk for mortality have not been studied. Thus, the aim of this study was to analyze the effect of hypokalemia on overall and cause-specific mortality.

**Design, Setting, Participants and Measurements:**

This is an analysis of BRAZPD II, a nationwide prospective cohort study. All patients on PD for longer than 90 days with measured serum potassium levels were used to verify the association of hypokalemia with overall and cause-specific mortality using a propensity match score to reduce selection bias. In addition, competing risks were also taken into account for the analysis of cause-specific mortality.

**Results:**

There was a U-shaped relationship between time-averaged serum potassium and all-cause mortality of PD patients. Cardiovascular disease was the main cause of death in the normokalemic group with 133 events (41.8%) followed by PD-non related infections, n=105 (33.0%). Hypokalemia was associated with a 49% increased risk for CV mortality after adjustments for covariates and the presence of competing risks (SHR 1.49; CI95% 1.01-2.21). In contrast, in the group of patients with K <3.5mEq/L, PD-non related infections were the main cause of death with 43 events (44.3%) followed by cardiovascular disease (n=36; 37.1%). For PD-non related infections the SHR was 2.19 (CI95% 1.52-3.14) while for peritonitis was SHR 1.09 (CI95% 0.47-2.49).

**Conclusions:**

Hypokalemia had a significant impact on overall, cardiovascular and infectious mortality even after adjustments for competing risks. The causative nature of this association suggested by our study raises the need for intervention studies looking at the effect of potassium supplementation on clinical outcomes of PD patients.

## Introduction

Potassium abnormalities are very common in dialysis patients, and both hyperkalemia and hypokalemia have been consistently associated with a high risk of all-cause and cardiovascular mortality [1]. Hypokalemia is found in approximately 35% of peritoneal dialysis (PD) patients [2–4]. The contribution of hypokalemia to the risk of mortality in PD patients is considerably higher than the one observed in hyperkalemia [5].

A large cohort study performed by Torlén et al., analyzing data of more than 120,000 dialysis patients (of which approximately 10,000 were on PD), observed that the population-attributable risk for all-cause mortality was 3.6% for hypokalemia and 1.9% for hyperkalemia [1]. This study raised some important and unanswered questions: first, does potassium fluctuation affect clinical outcomes? And second, is there a causal relationship or hypokalemia is merely a surrogate marker of malnutrition and other comorbidities?

Xu et al addressed the first question [6], in an analysis of 886 incident PD patients, demonstrating the effect of potassium variability (expressed as the within-patient standard deviation) on all-cause mortality. The authors concluded that higher serum potassium variability was associated with an independent increase in mortality risk, with the higher quartile presenting an adjusted hazard ratio of 2.43 (CI95% 1.03–5.46).

Nevertheless, the second question remains unanswered, since randomized clinical trials analyzing the causal relationship between hypokalemia and mortality would not be feasible due ethical reasons, and observational studies are associated with selection bias. One interesting approach to minimize the differences between groups would be a propensity match score. When used properly, the propensity match score improves considerably the balance of main characteristics between groups. Therefore, the aim of this study was to compare hypokalemic patients with patients with normal serum potassium levels in relation to all-cause, infectious and cardiovascular mortality in large Brazilian PD cohort using the propensity match score.

## Population and Methods

This is a nationwide prospective cohort study (BRAZPD II), launched in December 2004, which followed patients until January 2011, described in detail in previous publications [7]. The administrative structure of the BRAZPD II comprises a steering committee with 3 members, one project manager, one project coordinator and one biostatistician. The ethical committees of all participating centers approved the study. The list of all ethic review boards that approved the study can be found in (S1 File). All patients provided written consent, which was approved by the ethical committee and stored locally only in Portuguese. The database contains data from 122 dialysis centers of all regions from Brazil. Any person can submit a project to use data from BRAZPD II for analysis, under the supervision of the Steering Committee. The number of prevalent patients in each year corresponded to 65 to 70% of all PD patients in the country in that same year. Data collection included sociodemographic data, such as age (years), gender, race, cause of end-stage renal disease (ESRD), pre-dialysis care characteristics, family income, education level, distance from dialysis center, region where patients live and center experience in patient-year. Clinical data included body mass index (kg/m2), blood pressure (mmHg), presence of edema, and PD modality divided in Continuous Ambulatory Peritoneal Dialysis (CAPD) or Automated Peritoneal Dialysis (APD). APD is a term used to all forms of PD that employ a mechanized device to assist in the delivery and drainage of dialysate. The presence of comorbid conditions (lupus, malignancy, coronary artery disease, known left ventricular hypertrophy, stroke, peripheral artery disease and diabetes) was registered and the score of Davies calculated accordingly.

Brazil has a public health care system, which provides universal access to renal replacement therapy, and a group of patients start PD as first treatment. Currently, the prevalence of PD is similar to several countries from all continents including Europe, North-America (except Mexico) and Latin America.

**Fig 1:**
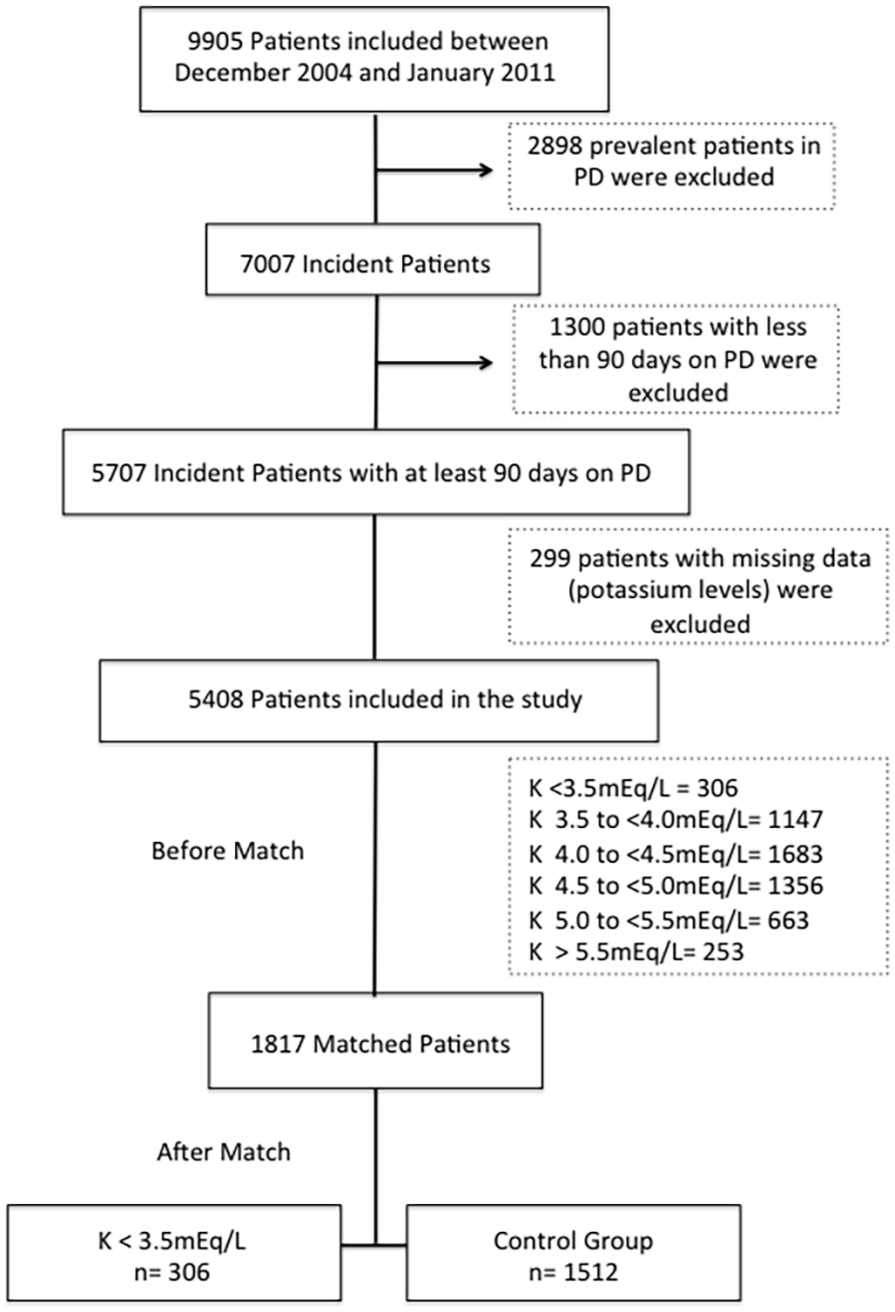
Study population.

Incident patients were defined as those individuals who started PD during the duration of the study, therefore being capture in the study since their first month on PD. All new patients on PD (incident patients) treated with PD for more than 90 days, and with available serum potassium concentrations in the database were included in the analysis (Fig 1). Patients were stratified in six groups according to baseline and time-averaged potassium levels according to the approach used in previous studies [1,6]: group I (<3.5mEq/L), group II (3.5 to <4.0mEq/L), group III (4.0 to <4.5mEq/L), group IV (4.5 to <5.0mEq/L), group V (5.0 to <5.5mEq/L) and group VI (≥5.5mEq/). Potassium measurement was performed monthly in all patients and throughout the whole duration of the observation period, according to the study protocol and aligned with the Brazilian regulatory rules for monitoring dialysis patients.

Patients on PD for less than 90 days were excluded to avoid the potential influence of prior therapies on clinical outcomes. To minimize the effect of the different prevalence of comorbidities across groups on patient survival, we matched patients from group I (hypokalemic patients) to subjects with normal potassium serum levels using several covariates and then compared groups as described below.

### Clinical outcomes

Outcomes were analyzed using the traditional Cox Proportional Hazards model and using the competing risk analysis proposed by Fine and Gray [8]. To be included in any multivariate analysis, the variables should have had a p value < 0.20 in the univariate analysis. Competing risks were defined as follows: (1) for overall mortality, any cause of drop out from therapy apart from death; (2) for cardiovascular (CV) mortality, any cause of drop out from therapy apart from death attributed to CV disease; and (3) for infectious mortality, any cause of drop out from therapy apart from death attributed to any kind of infection including peritonitis. All patients still alive at the end of the study were treated as censored.

### Matching procedure

A set of covariates was selected to estimate the propensity score. These were: age, body mass index (BMI), center experience, Davies score, diabetes, family income, gender, literacy, PD modality, race, previous hemodialysis (HD), duration of pre-dialysis care and year of initiation of PD. The propensity score (PS) was calculated using logistic regression, as proposed by Fine and Gray [8], and patients with potassium <3.5mEq/L (group I) were matched with controls using the nearest neighbor technique with a predefined caliper of 0.2. As sample size between the groups varies significantly, to optimize balance and precision, we matched patients using a ratio of 1:5 [9]. This matching procedure was done using the MatchIt package for R [10].

### Statistical analysis

Continuous variables were expressed as mean ± SD or median and range, while categorical variables (e.g., gender, race, primary renal disease, presence of comorbid conditions, initial therapy, current PD modality, etc) were expressed as frequencies or percentages. Cox proportional hazard models were estimated using SPSS 20.0 and sub-hazard distribution using competing risk analysis were calculated with the CRR function available in the CMPRSK package for R. Assumptions for proportional hazards and proportional sub-distribution hazards were checked with residual plots. Statistical significance was set at the level of p <0.05.

## Results

### Study population

From 9,905 patients, we included only incident patients patients, and excluded those on PD for less than 90 days and those missing serum potassium values. Of the remaining 5408 patients, we identified 306 with a time-averaged potassium < 3.5mEq/L, 1147 with 3.5 to <4.0mEq/L, 1683 with 4.0 to < 4.5mEq/L, 1356 with 4.5 to <5.0mEq/L, 663 with 5.0 to 5.5mEq/L and 253 ≥ 5.5mEq/L. After matching, there were 2 groups: 306 patients with K<3.5mEq/L and 1512 controls with normal potassium levels (Fig 1).

### Baseline characteristics

#### Entire cohort

The mean age of the entire study population (n = 5408) was 59±16 years, 52% were female, 75% had history of hypertension, 37% had history of previous hemodialysis, 63% were Caucasians, the prevalence of BMI < 18.5 was 6.4%, 42% were overweight (BMI > 25kg/m^2^) and diabetes was present in 44% of the patients. Baseline characteristics of the study population divided by sub-groups are presented in Table 1.

**Table 1:** Clinical and demographic characteristics by serum potassium.

Variable	Overall (n = 5408)	<3.5	3.5–≤ 4.0	4.0–≤4.5	4.5–≤ 5.0	5.0–≤ 5.5	> 5.5
**Age** > 65 years	59.3±15.93	62.9±16.2	60.7±16.6	59.7±16.0	58.9±15.3	56.3±15.3	55.5±14.52
	8.7%	50%	44%	40%	37%	28%	25%
**Diabetes**	44.7%	46.1%	42.0%	47.8%	43.0%	45.7%	42.3%
**Male gender**	47.6%	36.3%	46.5%	47.4%	49.0%	50.8%	52.2%
**Previous HD (Yes)**	37.0%	28.1%	33.5%	33.7%	39.7%	45.9%	47.4%
**Hypertension (yes)**	75.7%	70.3%	73.1%	75.4%	77.7%	76.0%	84.6%
**Pre-dialysis care (Yes)**		51.0%	55.4%	56.5%	49.2%	46.3%	39.1%
**BMI**							
<18.5	6.4%	6.5%	7.5%	6.5%	5.8%	6.3%	4.7%
18.5–24.9	51.8%	53.6%	51.9%	52.1%	52.5%	48.3%	52.2%
≥25	41.8%	39.9%	40.6%	41.4%	41.7%	45.4%	43.1%
**Davies Score**							
0	35.3%	29.7%	36.8%	33.2%	37.5%	35.9%	36.4%
1–2	58.7%	47.4%	40.6%	44.1%	39.2%	42.7%	40.3%
3–4	6.0%%	22.9%	22.6%	22.7%	23.3%	21.4%	23.3%
**Coronary artery disease**	22.1%	22.9%	21.5%	23.6%	22.5%	19.5%	17.8%
**Left Ventricular Hypertrophy**	30.0%	30.1%	33.4%	30.7%	29.9%	26.4%	19.4%
**Race**							
White	63.5%	66.3%	61.4%	63.6%	65.0%	64.4%	58.9%
**Primary renal disease**							
Diabetic nephropathy	38.2%	38.9%	35.8%	39.9%	37.4%	40.1%	36.0%
Hypertension	17.1%	19.3%	19.0%	15.4%	16.2%	16.6%	23.3%
Chronic Glomerulonephritis	9.7%	8.5%	8.6%	10.3%	10.4%	9.2%	9.5%
**Literacy**							
Up to 4 years	65.3%	73.9%	69.3%	62.3%	63.3%	64.6%	69.6%
**Center experience (patients-year)**	41.5±25.1	47.2±24.8	44.8±25.0	43.1±25.5	38.4±24.8	37.4±23.6	37.3±23.8

#### Matched patients

All variables were well balanced with the matching procedure (Table 2); the standardized differences of means between covariates can be seen in (S1 Table). There was no significant variability within patients before and after match (S1 and S2 Tables).

**Table 2:** Clinical and demographic characteristics after match.

Variable	Overall (n = 1817)	K<3.5mEq/L(n = 305)	Control group (n = 1512)	p
**Age** > 65 years	46.9%	62.9 ± 16.2	62.6 ±15.2	0.800.
		50.2%	46.3%	23
**Diabetes**	45.2%	45.9%	45.1%	0.42
**Male gender**	37.0%	36.4%	37.2%	0.84
**Previous HD** (Yes)	27.5%	28.2%	27.4%	0.78
**Hypertension** (yes)	71.3%	70.5%	71.4%	0.73
**Pre-dialysis care** (Yes)	50.5%	50.8%	50.5%	0.95
**BMI**				0.73
<18.5	5.8%	6.6%	5.7%	
18.5–24.9	52.8%	53.8%	52.6%	
≥25	41.3%	39.7%	41.7%	
**Coronary Artery Disease**	22.4%	22.6%	22.3%	0.88
**Left Ventricular Hypertrophy**	31.3%	30.2%	31.5%	0.68
**Davies Score**				0.42
0	33.2%	29.9%	33.9%	
1–2	42.7%	64.5%	59.5%	
3–4	24.1%	5.6%	6.6%	
**Race**				
White	65.8%	66.6%	65.7%	0.79
**Primary renal disease**				0.81
Diabetic nephropathy	37.8%	39.0%	37.5%	
Hypertension	17.4%	19.3%	17.1%	
Chronic Glomerulonephritis	8.9%	8.6%	9.0%	
**Education**				
(Up to 4 years)	27.4%	26.2%	27.6%	0.67
**Center experience** (patients-year)	47.7 ±25.2	47.1 ±24.8	47.9 ±25.3	0.63

### Clinical outcomes

#### Entire cohort

Out of a total of 5,408 patients, 1,026 died during the follow up. Cardiovascular disease was the leading cause of death with 450 events (43.9%), followed by PD-non related infections with 351 events (34.2%). Peritonitis–related deaths corresponded to 95 events (9.2%) and 135 were spread between others or unknown causes.

#### Matched patients

There were 415 fatal events during the study period: 318 in the control group and 97 in patients with K<3.5mEq/L. Cardiovascular disease was the main cause of death in the normokalemic group with 133 events (41.8%) followed PD-non related infections, n = 105 (33.0%). In contrast, in the group of patients with K <3.5mEq/L, PD-non related infections were the main cause of death with 43 events (44.3%) followed by cardiovascular disease (n = 36; 37.1%). Deaths related to peritonitis were 10.7% in the control group (n = 34) and 7.2% in the group with hypokalemia (n = 7).

### Overall mortality

#### Entire cohort

**Fig 2:**
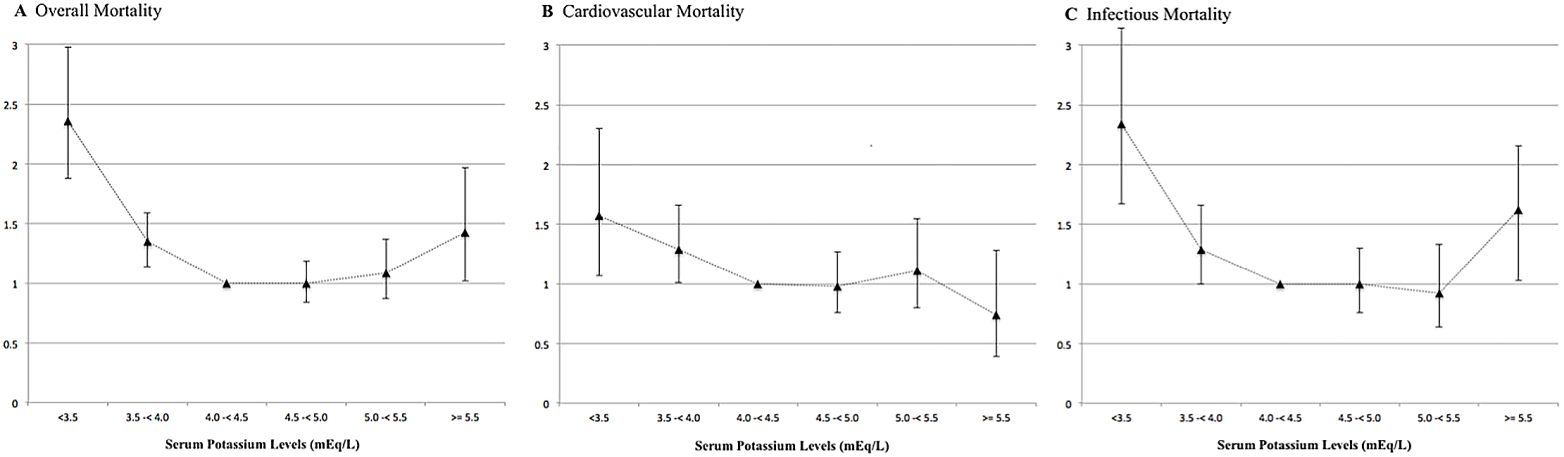
All-cause and cause-specific mortality rates for the entire population (n = 5408) using a competing risk analysis.

Fig 2 shows an U-shaped relationship between time-averaged serum potassium levels and all-cause mortality. Compared to the reference of 4.0 to 4.5mEq/L, patients from group I (K<3.5mEq/L) had a HR 2.36 (CI95%1.88–2.98) and patients from group VI (K>5.5mEq/L) had a HR 1.43 (CI95%1.02–2.00). These associations were not found when groups were stratified using baseline potassium. Patients with lower potassium levels presented a higher prevalence of comorbidities including higher age, Davies Score and low BMI (Table 1). Supplementary data presents information and results of the whole model. (S3 Table)

#### Matched patients

**Fig 3:**
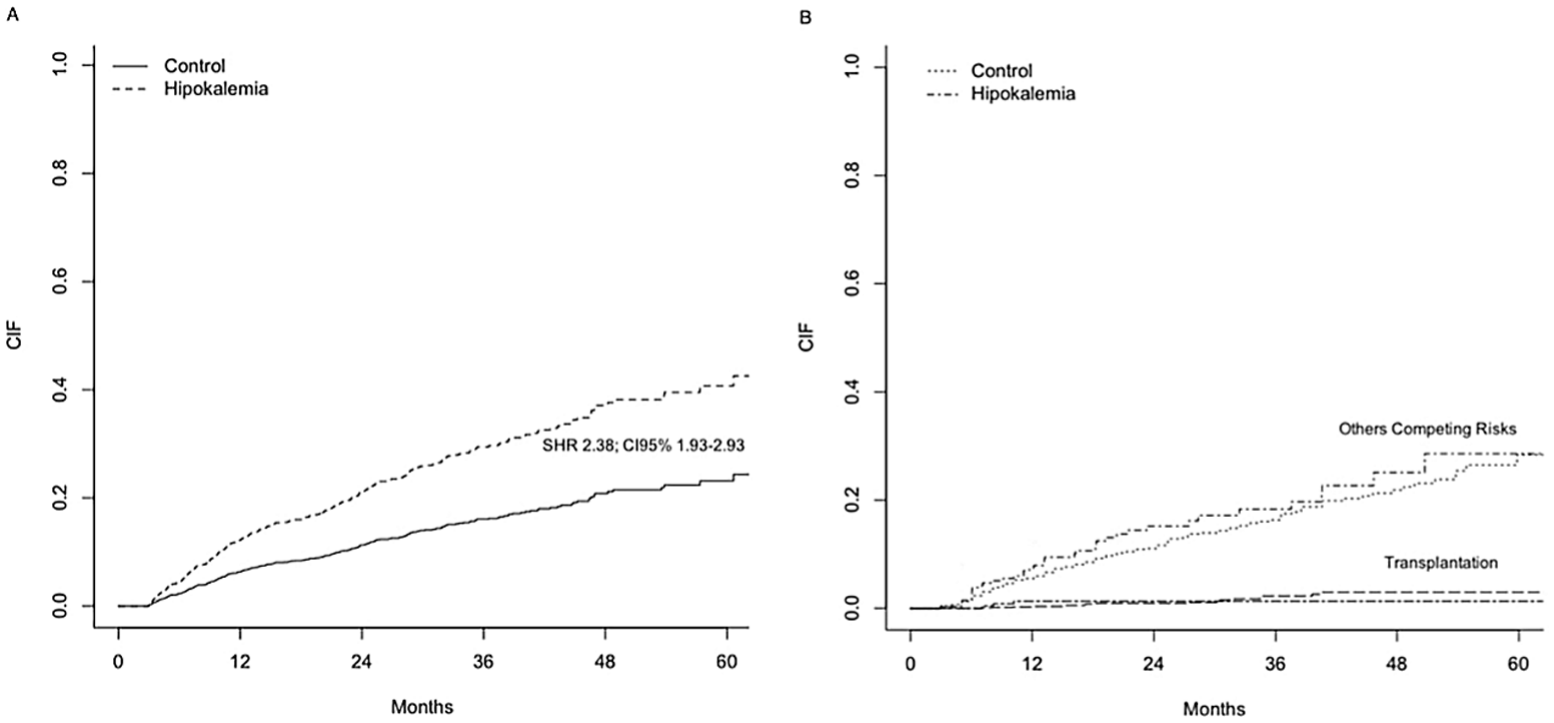
All-Cause Mortality for Matched Patients. Cumulative incidence failure (CIF) for the primary event of interest (A) and the competing risks (B); SHR: Sub-distribution Hazard Ratio; CI: Confidence Interval.

Covariates were very well balanced after match and the comparison of general characteristics of both groups can be seen in Table 2. On multivariate Cox analysis, low serum potassium level remained a significant risk factor for all-cause mortality after adjusting for covariates (HR2.00; CI95% 1.59–2.52). Taking competing risks into account, the association of hypokalemia with mortality was even higher (SHR2.38; CI95% 1.93–2.93). Fig 3 shows the cumulative incidence curves of all-cause mortality for the primary event of interest and the competing risks.

### Cardiovascular mortality

#### Entire cohort

Compared with the reference levels of 4.0 to 4.4mEq/L, potassium serum levels below 4.0mEq/L were associated with cardiovascular mortality: <3.5mEq/L (SHR 1.57; CI95% 1.07–2.30) and 3.5 to 3.9mEq/L (SHR1.29; CI95%1.01–1.66). Others independent risk factors were: age>65 years, no pre-dialysis care, diabetes, history of previous hemodialysis, coronary artery disease and left ventricular hypertrophy. Details about all risk factors can be found in (S4 Table).

#### Matched patients

**Fig 4:**
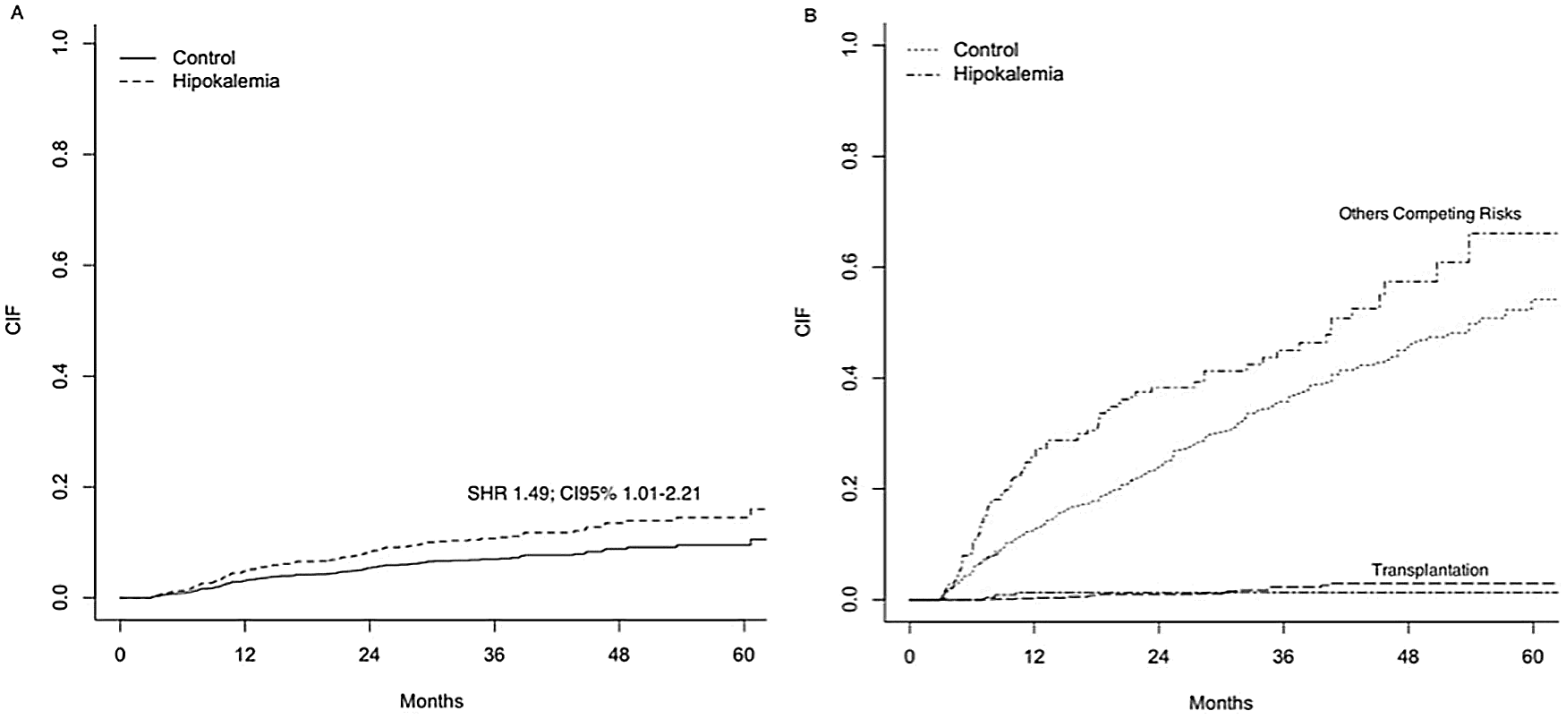
Cardiovascular Mortality for Matched Patients. Cumulative incidence failure (CIF) for the primary event of interest (A) and the competing risks (B); SHR: Sub-distribution Hazard Ratio; CI: Confidence Interval.

Hypokalemia was associated with a 49% increased risk for CV mortality after adjustments for covariates and the presence of competing risks (SHR 1.49; CI95% 1.01–2.21). Fig 4 shows the cumulative incidence failure (CIF) for the event of interest (A) and the competing risks (B). Others independent risk factors were: age>65 years, diabetes, PD modality and coronary artery disease. Details about all risk factors can be found in (S5 Table).

### Infectious mortality

#### Entire cohort

Serum potassium levels below 4.0mEq/L were also a risk factor for PD-non related infectious mortality compared to the reference level of 4.0 to 4.4mEq/L: <3.5mEq/L (SHR 2.34; CI95% 1.67–3.29) and 3.5 to 3.9mEq/L (SHR 1.29; CI95% 1.01–1.66). Other independent risk factors were: age>65 years, no pre-dialysis care, diabetes, literacy below four years, BMI < 18.5 and previous hemodialysis. When only non-PD related infections the SHR was 2.65 (CI95%1.84–3.84). For peritonitis there was no association between any serum potassium levels with patient survival (for K<3.5mEq/L: SHR1.22; CI95% 0.52–2.83). Details about all risk factors can be found in (S6 Table).

#### Matched patients

**Fig 5:**
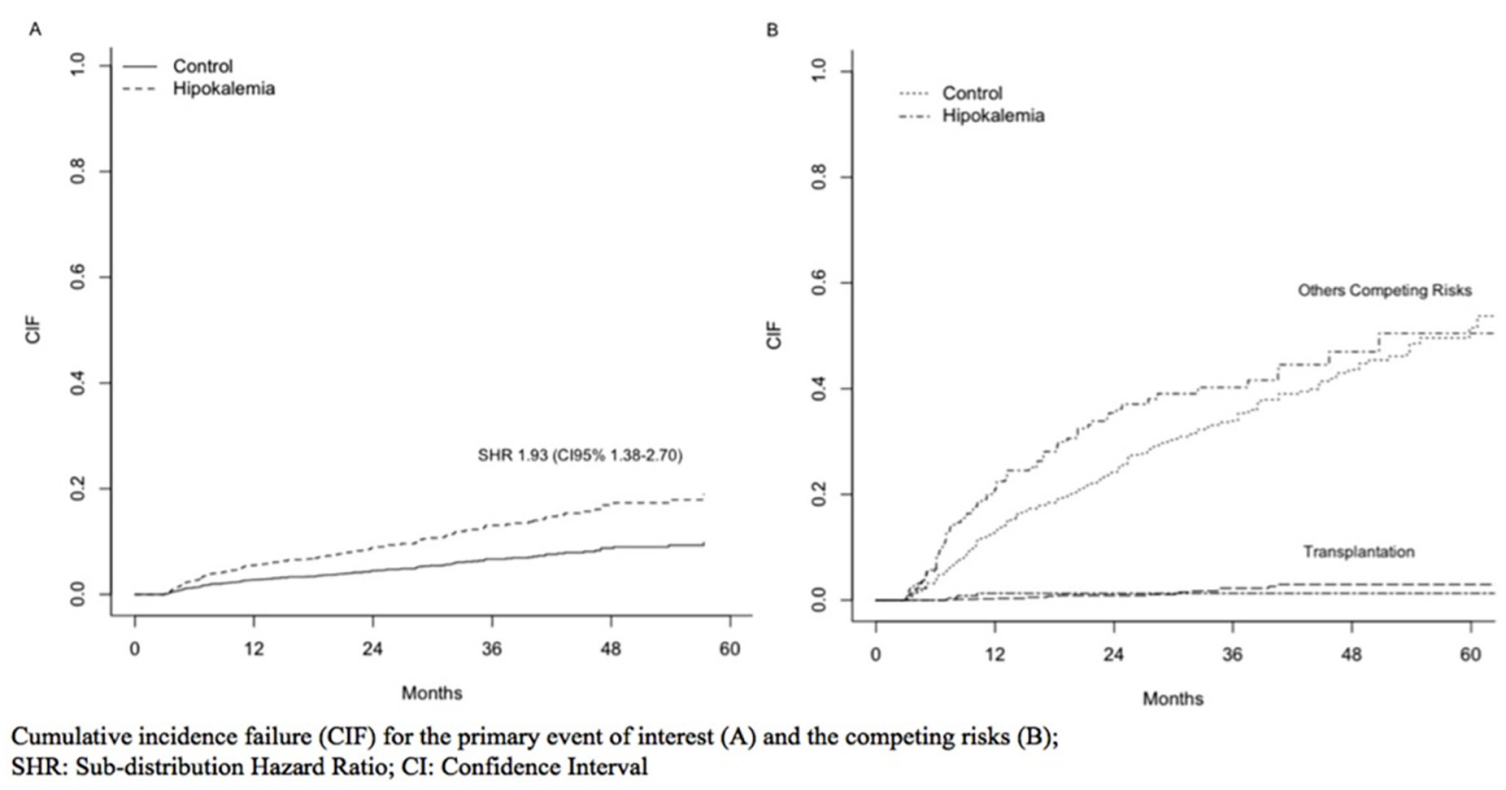
Infectious Mortality for Matched Patients. Cumulative incidence failure (CIF) for the primary event of interest (A) and the competing risks (B); SHR: Sub-distribution Hazard Ratio; CI: Confidence Interval.

The risk for death by infectious causes was greater for patients with low time-averaged serum potassium levels, with a SHR of 1.93 (CI95% 1.38–2.70). Others independent risk factors were: age>65 years, no pre-dialysis care and BMI<18.5. For the specific causes of PD-non related infections the SHR was 2.19 (CI95% 1.52–3.14) while for peritonitis was SHR 1.09 (CI95% 0.47–2.49). Details about all risk factors can be found in (S7 Table). Fig 5 shows the CIF for the event of interest (A) and the competing risks (B). Hypokalemia present a higher risk for time to first peritonitis episode (S8 and S9 tables).

## Discussion

This is the first large cohort study bias using a propensity score to match hypokalemic patients with controls to reduce selection in the analysis of hypokalemia as a cause of mortality, and not just a surrogate marker. Overall, our results showed that patients with hypokalemia have a higher risk of death by cardiovascular, infectious and all-cause mortality, even after considering the presence of competing risks and adjustments for several covariates.

Hypokalemia is a common condition in PD, and according to previous reports its prevalence can reach 36% [1,2,4,11]. In our study the prevalence of hypokalemia (K< 3,5 mEq/L) was 5.6%, similar to values observed in large contemporary cohorts [1]. Hypokalemia was significantly more prevalent in females, elderly, patients with low education level and in those with a higher prevalence of comorbidities. These findings were expected and may partially justify the association of hypokalemia and poor clinical outcomes during the past decades that include not only a higher risk for peritonitis, but also higher mortality rates [1,2,12]. Recently, a large cohort study confirmed the impact of hypokalemia on patients’ survival. Analyzing more than 10.000 PD patients, Tórlen et al reported a 51% increased risk for all-cause mortality in individuals with K<3.5mEq/L. Nevertheless, hypokalemia is frequently associated with comorbidities that are known risk factors for poor outcomes such as malnutrition and inflammation [2], and an important question remains unanswered: despite the good quality of the previous studies, we do not know whether or not hypokalemia has a direct effect in mortality rates or is just a surrogate marker of comorbidities [5]. However, it is important to note that given the observational design of all these studies, including the present, this issue is not easy to address. To overcome this limitation, we used a propensity score to mimic some of the characteristics of randomized clinical trials (RCT) instead of relying on the use of regression adjustments to account for differences at the baseline [13]. After matching for several covariates we obtained a good balance among variables, and therefore our study provides more robust evidence. This approach does not substitute a RCT, but provides significant approximation in terms of group balance.

Our findings confirm the association between hypokalemia and poor patient survival, and strongly suggest a causal relationship due the particular statistical design used for this analysis. However, it is important to emphasize that our study, similarly to all other large cohort studies, does not have data related to malnutrition and inflammation, such albumin, CRP, IL-6 or any other marker. So, we cannot discard the possibility that the group with hypokalemia were different in terms of such markers after match.

To better understand the potential mechanisms behind this association, we further analyzed cause specific mortality in our cohort, refining the approach used by Torlén et al. Traditional methods of survival analysis commonly censor all causes of competing events. Such statistical approach commonly yields to overestimation of cumulative mortality probabilities for each of the separate causes of death [8,14]. Therefore, distinctly from previous studies, we considered the presence of competing risks throughout the analysis. In line with the findings of Torlén et al, in the present study hypokalemia was also an important risk factor for cardiovascular, infectious and all-cause mortality. Interestingly, an analysis of a group of patients with chronic heart failure and no ESRD found hypokalemia as a risk factor for mortality using similar statistical methods, reinforcing our statement that hypokalemia may play a direct role in the increased risk of mortality [15]. Of note, hyperkalemia was not significantly associated with CV mortality in our analysis. This finding can be explained by the small prevalence of hyperkalemia in our cohort, particularly in very high levels.

One of the most interesting finding of the study by Torlén et al was the association between hypokalemia with infectious mortality. Unfortunately, the authors didn't report a subgroup analysis according to the source of infection. Our results were similar, but we advance with the analysis of infections related and not related to PD. To the best of our knowledge, this is the first time that this stratification was performed in a large cohort study. The importance of this approach is to better understand the underlying mechanisms associating hypokalemia and death according to infection site. The fact that hypokalemia was an independent risk factor even after matching for several important comorbidities reduce the possibility of hypokalemia was acting only as a surrogate marker for others comorbidities.

It is known that PD-related peritonitis is commonly associated with low mortality rates, usually below 5% [16]. In our study, hypokalemia did not influence mortality related to peritonitis. In contrast, it was strongly related to mortality caused by infections not related to PD. This finding suggests that hypokalemia may play a role in the hemodynamic hyporesponsiveness that occur in some patients with septic shock. Data from animal models suggest that low potassium, even when within the conventional range of serum potassium, may impair myocardial contractile and relaxation response to epinephrine [17].

This study presents some limitations: (I) although matched and well balanced for several covariates using a propensity score approach, this method does not account for unmeasured confounders unlike a randomized controlled clinical trial; II) the absence of data related to residual renal function, although hyperkalemia and not hypokalemia are more likely to be present in anuric patients; (III) the particular causes of CV death were not captured in the study, and mortality related to vascular or myocardial causes cannot be differentiated and finally (IV) even after the matching procedure, which included BMI, the lack of data related to malnutrition and other inflammatory markers can not rule out the possibility of differences in these markers between groups. On the other hand, our study has important strengths: (I) it was based on a large national prospective cohort, with all races and regions well represented and with a standardized data collection providing a good external validity; (II) the groups were well balanced for several clinical and demographic variables using a propensity score analysis; (III) and finally, competing risks were taken into account in all analysis.

In conclusion, hypokalemia was significantly associated with higher risk of cardiovascular, infectious and all-cause mortality, even after adjustments for competing risks and with well-balanced groups. The underlying mechanisms behind the association of hypokalemia and infectious mortality deserve further investigation. Finally, our results raise the need for future intervention studies looking at the effect of hypokalemia correction on patient survival.

## Supporting Information

S1 Table Potassium variability—Overall population. (DOCX)

S2 Table Potassium variability—Matched patients. (DOCX)

S3 Table Risk factors for overall mortality. (DOCX)

S4 Table Risk factors for cardiovascular mortality. (DOCX)

S5 Table Risk factors for cardiovascular mortality in the matched cohort. (DOCX)

S6 Table Risk factors for infectious mortality. (DOCX)

S7 Table Independent risk factors for infectious mortality in the matched cohort. (DOCX)

S8 Table Time to first peritonitis episode—Overall population. (DOCX)

S9 Table Time to first peritonitis episode—Matched patients. (DOCX)

S1 File Ethic Review Boards that approved the study. (DOCX)
