# Supplementary material for: Low Serum Potassium Levels Increase the Infectious-Caused Mortality in Peritoneal Dialysis Patients: A Propensity-Matched Score Study
Source: PLoS One. 2015 Jun 19;10(6):e0127453. doi: 10.1371/journal.pone.0127453 (PMC4474697; doi:10.1371/journal.pone.0127453)
Supplement: S1 Table — (DOCX) [file pone.0127453.s001.docx]

**S1 Table. Potassium variability - Overall population**

|  | **<3.5** | **3.5 to <4.0** | **4.0 to <4.5** | **4.5 to <5.0** | **5.0 to < 5.5** | **> 5.5** |
| --- | --- | --- | --- | --- | --- | --- |
| **Standard deviation** | 0.43 | 0.42 | 0.43 | 0.46 | 0.49 | 0.53* |
| **Coefficient of variability** | 0.13 | 0.10 | 0.11 | 0.10 | 0.09 | 0.09 |

*** p< 0.05**
