# Supplementary material for: Low Serum Potassium Levels Increase the Infectious-Caused Mortality in Peritoneal Dialysis Patients: A Propensity-Matched Score Study
Source: PLoS One. 2015 Jun 19;10(6):e0127453. doi: 10.1371/journal.pone.0127453 (PMC4474697; doi:10.1371/journal.pone.0127453)
Supplement: S2 Table — (DOCX) [file pone.0127453.s002.docx]

**S2 Table. Potassium variability - Matched patients**

|  | **Normokalemia** | **< 3.5mEq/L** |
| --- | --- | --- |
| **Standard deviation** | 0.44 | 0.43 |
| **Coefficient of variability** | 0.10 | 0.13 |
