# Supplementary material for: Low Serum Potassium Levels Increase the Infectious-Caused Mortality in Peritoneal Dialysis Patients: A Propensity-Matched Score Study
Source: PLoS One. 2015 Jun 19;10(6):e0127453. doi: 10.1371/journal.pone.0127453 (PMC4474697; doi:10.1371/journal.pone.0127453)
Supplement: S3 Table — (DOCX) [file pone.0127453.s003.docx]

**S3 Table. Risk factors for overall mortality**

| **Variables** | **HR (CI95%)** |
| --- | --- |
| Age > 65 years | 2.43 (2.14-2.76) |
| Diabetes (yes) | 1.65 (1.46-1.88) |
| Modality (APD) | 0.76 (0.66-0.88) |
| Race (Black) | 0.83 (0.73-0.95) |
| Literacy (>4 years of formal education) | 0.80 (0.69-0.92) |
| Body mass index < 18.5 | 1.67 (1.33-2.09) |
| Previous hemodialysis (yes) | 1.33 (1.17-1.51) |
