# Supplementary material for: Low Serum Potassium Levels Increase the Infectious-Caused Mortality in Peritoneal Dialysis Patients: A Propensity-Matched Score Study
Source: PLoS One. 2015 Jun 19;10(6):e0127453. doi: 10.1371/journal.pone.0127453 (PMC4474697; doi:10.1371/journal.pone.0127453)
Supplement: S4 Table — (DOCX) [file pone.0127453.s004.docx]

**S4 Table. Risk factors for cardiovascular mortality**

| **Variables** | **HR (CI95%)** |
| --- | --- |
| Center experience in patient year | 0.995 (0.991-0.999) |
| Diabetes (yes) | 1.75 (1.44-2.12) |
| Previous hemodialysis (yes) | 1.31 (1.08-1.58) |
| Pre-dialysis care (yes) | 0.77 (0.63-0.92) |
