# Supplementary material for: Low Serum Potassium Levels Increase the Infectious-Caused Mortality in Peritoneal Dialysis Patients: A Propensity-Matched Score Study
Source: PLoS One. 2015 Jun 19;10(6):e0127453. doi: 10.1371/journal.pone.0127453 (PMC4474697; doi:10.1371/journal.pone.0127453)
Supplement: S5 Table — (DOCX) [file pone.0127453.s005.docx]

**S5 Table. Risk factors for cardiovascular mortality in the matched cohort**

| **Variables** | **SHR (CI95%)** |
| --- | --- |
| Age > 65 years | 2.54 (1.82-3.56) |
| Diabetes (yes) | 1.65 (1.21-2.26) |
| PD modality (APD) | 0.59 (0.39-0.89) |
| Coronary artery disease (no) | 0.46 (0.33-0.62) |
