# Supplementary material for: Low Serum Potassium Levels Increase the Infectious-Caused Mortality in Peritoneal Dialysis Patients: A Propensity-Matched Score Study
Source: PLoS One. 2015 Jun 19;10(6):e0127453. doi: 10.1371/journal.pone.0127453 (PMC4474697; doi:10.1371/journal.pone.0127453)
Supplement: S6 Table — (DOCX) [file pone.0127453.s006.docx]

**S6 Table. Risk factors for infectious mortality**

| **Variables** | **HR (CI95%)** |
| --- | --- |
| Diabetes (yes) | 1.42 (1.17-1.72) |
| Modality (APD) | 0.79 (0.63-0.99) |
| Literacy (>4 years) | 0.72 (0.57-0.89( |
| Body mass index < 18.5 | 2.26 (1.66-3.07) |
| Previous Hemodialysis (yes) | 1.29 (1.06-1.57) |
