# Supplementary material for: Low Serum Potassium Levels Increase the Infectious-Caused Mortality in Peritoneal Dialysis Patients: A Propensity-Matched Score Study
Source: PLoS One. 2015 Jun 19;10(6):e0127453. doi: 10.1371/journal.pone.0127453 (PMC4474697; doi:10.1371/journal.pone.0127453)
Supplement: S7 Table — (DOCX) [file pone.0127453.s007.docx]

**S7 Table. Independent risk factors for infectious mortality in the matched cohort**

| **Variables** | **SHR (CI95%)** |
| --- | --- |
| Age > 65 years | 1.95 (1.45-2.62) |
| Pre-dialysis care (no) | 1.45 (1.07-1.95) |
| Diabetes (yes) | 1.37 (1.02-1.86) |
| Body mass index < 18.5 | 2.35 (1.47-3.75) |
