# Supplementary material for: Low Serum Potassium Levels Increase the Infectious-Caused Mortality in Peritoneal Dialysis Patients: A Propensity-Matched Score Study
Source: PLoS One. 2015 Jun 19;10(6):e0127453. doi: 10.1371/journal.pone.0127453 (PMC4474697; doi:10.1371/journal.pone.0127453)
Supplement: S8 Table — (DOCX) [file pone.0127453.s008.docx]

**S8 Table. Time to first peritonitis episode - Overall population**

|  | **<3.5** | **3.5 to <4.0** | **4.0 to <4.5** | **4.5 to <5.0** | **5.0 to < 5.5** | **> 5.5** |
| --- | --- | --- | --- | --- | --- | --- |
| **Hazard ratio (CI95%)** | 1.72 (1.38-2.14) | 1.34(1.17-1.53) | Reference | 0.96(0.83-1.11) | 0.88(0.73-1.06) | 1.24(0.95-1.62) |
