# Supplementary material for: Low Serum Potassium Levels Increase the Infectious-Caused Mortality in Peritoneal Dialysis Patients: A Propensity-Matched Score Study
Source: PLoS One. 2015 Jun 19;10(6):e0127453. doi: 10.1371/journal.pone.0127453 (PMC4474697; doi:10.1371/journal.pone.0127453)
Supplement: S9 Table — (DOCX) [file pone.0127453.s009.docx]

**S9 Table. Time to first peritonitis episode - Matched patients**

|  | **Normokalemia** | **< 3.5mEq/L** |
| --- | --- | --- |
| **Hazard ratio (CI95%)** | Reference | 1.56(1.25-1.95) |
