# Supplementary material for: Low Serum Potassium Levels Increase the Infectious-Caused Mortality in Peritoneal Dialysis Patients: A Propensity-Matched Score Study
Source: PLoS One. 2015 Jun 19;10(6):e0127453. doi: 10.1371/journal.pone.0127453 (PMC4474697; doi:10.1371/journal.pone.0127453)
Supplement: S1 File — (DOCX) [file pone.0127453.s010.docx]

**S10 File.**

**Ethic Review Boards that approved the study:**

- Ameneg - Assistência Médica Nefrológica de Guarulhos
- Associaçao Hospital de Bauru
- Biocor - Hospital de Doencas Cardiologicas
- Casa de Saude e Maternidade Nossa Senhora do Perpétuo Socorro
- Clínica de Doenças Renais - Curitiba
- Clínica de Doenças Renais - Goiania
- Clínica de Doenças Renais - Imperatriz
- Clínica de Doenças Renais - Sao Jose Pinhais
- CDTR_Centro Dialise Transplante Renal
- Centro Nefrologia Teresopolis
- Centro Nefrologico Minas Gerais
- Centro Trat. Doencas Renais Joinville
- Centro Tratamento Renal Zona Sul
- Clinica de Nefrologia Santa Teresa - Rio De Janeiro
- CLINEPA Clinica de Nefrologia da Paraiba
- Clines
- Clinica de Nefrologia do Sergipe - CLINESE
- Clinica do Rim do Carpina
- Clinica Evangelico S/C Ltda
- Clinica Nefrologia de Franca
- Clinica de Nefrologia Santa Rita
- Clinica de Nefrologica Sao Goncalo
- Clinica Paulista de Nefrologia
- Clinica Renal Manaus
- Clinica Senhor Do Bonfim
- Clinica Senhor Do Bonfim Ltda Filial
- Clinica Tratamento Renal
- Cuiaba Cenec
- Clire Clinica de Doencas Renais
- Famesp Botucatu
- UNICAMP - Universidade Estadual de Campinas
- Hospital de Clinicas da Faculdade de Medicina da Universidade de São Paulo
- Fundaçao Civil Santa Casa de Misericórdia de Franca
- Fundaçao Inst ituto Mineiro Estação de Pesquisa Nefrológicas
- Gamen - Rio de Janeiro
- Hospital de Base
- Histocom Sociedade Civil Ltda
- Hospital Universitário Prof. Edgard Santos
- Hospital Beneficência Portuguesa de Pernambuco
- Hospital Cidade Passo Fundo
- Hospital e Maternidade Angelina Caron
- Hospital Evangelico Vila Velha
- Hospital Geral Bonsucesso
- Hospital Geral de Goiania
- Hospital Infantil Joana de Gusmao
- Hospital São Joao Deus
- Hospital São Jorge
- Hospital São Jose do Avai
- Hospital Sao Vicente de Paula -João Pessoa
- Hospital Sao Vicente de Paulo
- Hospital Servidor do Estado - Ipase
- Hospital Universitário Presidente Dutra - Maranhão
- Hospital Universitario Antonio Pedro
- Hospital Vita Volta Redonda S/A
- Pontifícia Universidade Católica do Paraná
- Universidade Federal de Goias
- Hospital do Servidor Público Estadual - Sao Paulo
- Imip
- Instituto Capixaba de Doencas Renais
- Instituto Capixaba de Doencas Renais - Cariacica
- Instituto Capixaba de Doencas Renais - Serra
- Instituto do Rim de Fortaleza
- Instituto do Rim de Marilia
- Instituto do Rim do Parana S/C Ltda
- Instituto do Rim Santo Antonio da Platina
- Instituto de Hemodialise de Sorocaba
- Instituto de Medicina Nuclear Endocrina
- Instituto de Nefrologia de Mogi Das Cruzes
- Instituto de Nefrologia de Suzano
- Instituto de Nefrologia Souza e Costa
- Instituto de Urologia e Nefrologia Barra Mansa
- Instituto de Urologia e Nefrologia de São José do Rio Preto
- Medservsp
- Nefrocentro
- Nefroclinica Caxias do Sul
- Nefroclinica Foz do Iguacu
- Nefroclinica Uberlandia
- Nefron Clinica Natal
- Nefron Contagem
- Nephron Pelotas
- Nephron São Paulo
- Nucleo Nefrologia Belo Horizonte
- Pro Nephron
- Prorim Campos dos Goitacazes
- Pontifícia Universidade Católica de Porto Alegre
- Renalcare Serviços Medicos Ltda
- Renalcor Angra dos Reis
- Renalcor Rio de Janeiro
- Renalvida, Rien Rio de Janeiro
- Santa Casa de Adamantina
- Santa Casa de Jau Unefro
- Santa Casa De Marilia
- Santa Casa de Ourinhos
- Santa Casa de Santo Amaro,
- Santa Casa de Sao Jose Dos Campos
- Santa Casa de Votuporanga
- Serviço de Nefrologia de Ribeirao Preto
- Universidade Estadual do Rio de Janeiro
- Uni Rim Joao Pessoa
- Unidade de Nefrologia de Assis
- Unirim Unidade de Doenças Renais
- Unirim Unidade Renal do Portao
- UNTR Unidade de Nefrologia e Transplante.
